# Global Habitat Suitability for Framework-Forming Cold-Water Corals

**DOI:** 10.1371/journal.pone.0018483

**Published:** 2011-04-15

**Authors:** Andrew J. Davies, John M. Guinotte

**Affiliations:** 1 School of Ocean Sciences, Bangor University, Menai Bridge, Anglesey, United Kingdom; 2 Marine Conservation Biology Institute, Bellevue, Washington, United States of America; University of Glamorgan, United Kingdom

## Abstract

Predictive habitat models are increasingly being used by conservationists, researchers and governmental bodies to identify vulnerable ecosystems and species' distributions in areas that have not been sampled. However, in the deep sea, several limitations have restricted the widespread utilisation of this approach. These range from issues with the accuracy of species presences, the lack of reliable absence data and the limited spatial resolution of environmental factors known or thought to control deep-sea species' distributions. To address these problems, global habitat suitability models have been generated for five species of framework-forming scleractinian corals by taking the best available data and using a novel approach to generate high resolution maps of seafloor conditions. High-resolution global bathymetry was used to resample gridded data from sources such as World Ocean Atlas to produce continuous 30-arc second (∼1 km^2^) global grids for environmental, chemical and physical data of the world's oceans. The increased area and resolution of the environmental variables resulted in a greater number of coral presence records being incorporated into habitat models and higher accuracy of model predictions. The most important factors in determining cold-water coral habitat suitability were depth, temperature, aragonite saturation state and salinity. Model outputs indicated the majority of suitable coral habitat is likely to occur on the continental shelves and slopes of the Atlantic, South Pacific and Indian Oceans. The North Pacific has very little suitable scleractinian coral habitat. Numerous small scale features (i.e., seamounts), which have not been sampled or identified as having a high probability of supporting cold-water coral habitat were identified in all ocean basins. Field validation of newly identified areas is needed to determine the accuracy of model results, assess the utility of modelling efforts to identify vulnerable marine ecosystems for inclusion in future marine protected areas and reduce coral bycatch by commercial fisheries.

## Introduction

One of the most enigmatic groups of deep-sea organisms are framework-forming cold-water corals. Compared to many other deep-sea ecosystems, cold-water corals are relatively well researched, but still face significant threats from human activities [1], [2], [3]. Their susceptibility to anthropogenic impacts and slow rates of recovery from disturbance has led to an increasing awareness that cold-water coral ecosystems deserve full protection both within countries' Exclusive Economic Zones (EEZs) and on the high seas. Predictive habitat modelling is increasingly being used as a cost effective tool to identify where vulnerable marine ecosystems (VMEs) could occur and to provide insight into the environmental drivers that control their distribution [4], [5], [6], [7]. The enormous costs associated with the operation of remotely operated vehicles (ROVs), submersibles, and ships with multibeam capability reinforce the need for well developed predictive habitat models to guide research, conservation and management initiatives. Refined models can be used to target areas with the highest probability of discovering cold-water coral ecosystems and contribute to the establishment of ecologically coherent networks of Marine Protected Areas (MPAs).

In this manuscript, ‘reefs’ are defined as biogenic structures formed by azooxanthellate scleractinian corals that alter sediment deposition, provide complex structural habitat and are subject to periodic growth and (bio)erosion [2], [3]. Cold-water coral reefs can be many meters in height, kilometres in length and provide important habitat and nursery areas for many species, including commercially important fish species [2]. Six species of Scleractinia are known to form reef frameworks in the deep sea, *Enallopsammia rostrata*, *Goniocorella dumosa*, *Lophelia pertusa*, *Madrepora oculata*, *Oculina varicosa* and *Solenosmilia variabilis* [8]. There has been significant bias towards *L. pertusa* particularly with respect to research and management, driven in part by the extent, accessibility and it's prominence as a flagship species for deep-sea conservation [1]. This is especially evident in the North Atlantic, where *L. pertusa* is the dominant framework-forming species. It is important to note that while the other five species are not as well studied as *L. pertusa*, they are the dominant framework-forming scleractinians in many regions of the world's oceans (i.e. the South Pacific). *Madrepora oculata* often occurs as a secondary framework-former growing in tandem with species such as *L. pertusa* and *G. dumosa* [8]. Similarly, *E. rostrata* is also found associated with *L. pertusa*, *M. oculata* and/or *S. variabilis* and can form massive dendroid colonies up to 1 m thick [8]. *Oculina varicosa* is unusual in that it is found both in shallow waters (<50 m with algal symbionts) and in the deep sea where it is azoozanthellate (depth range is ∼50 to 100 m). In the deep sea, *O. varicosa* forms tall, fragile reefs that can reach 35 m in height [9]. *Goniocorella dumosa* is mostly found in the southern hemisphere and is a dominant framework-former in New Zealand waters [10]. *Solenosmilia variabilis* has a cosmopolitan distribution, but has not been found in the Antarctic, north Pacific or east Pacific [10].

There are several characteristics of these framework-forming cold-water corals that make them vulnerable to a range of anthropogenic impacts. Their slow growth rate, fragility, and longevity (i.e. some *L. pertusa* reefs in the North Atlantic were dated to 4550 years old [11]) make them particularly vulnerable to human activities including: bottom-contact fishing activity [11], [12], hydrocarbon extraction and exploration [3] and the emerging threat of seabed mining [13]. Perhaps more severe, is the threat ocean acidification poses to cold-water coral reef ecosystems [14]. Such a pervasive range of impacts and the long recovery periods of these organisms has led to global efforts to conserve these ecosystems. One such mechanism was put forward in 2006 by the United Nations General Assembly, calling upon member states and regional fisheries management organisations to halt bottom-contact fishing in high seas areas where seamounts, hydrothermal vents, and cold-water corals are known or are likely to occur based on the best available scientific information (UN GA, Draft resolution of the 61^st^ session of the General Assembly, 6^th^ December 2006, A/61/L.38). Participating states could not reach consensus on the bottom trawling moratorium, but there is growing international support for habitat suitability modelling in the deep sea.

Several studies have been conducted that utilise a range of different presence-only habitat suitability modelling techniques on deep-sea species [4], [5], [6], [7], [15], [16]. However, there have been no major developments in addressing many of the current limitations of predictive modelling in the deep sea. The lack of environmental data at high resolutions is perhaps the greatest limitation to deep-sea modelling efforts [4], [6]. To address this, studies have focused on improving local-scale habitat suitability modelling by integrating digital terrain variables derived from multibeam bathymetry [5], [16], [17]. Whilst this approach produces valuable data on species distributions in localised areas (1–100 km^2^), it requires intensive and often expensive sampling techniques be conducted prior to modelling the area (e.g. multibeam bathymetry and video surveys). As such, local approaches are of limited impact in the broadscale identification of unknown habitat for cruise planning, management and conservation initiatives. Regional and global scale models are needed to predict habitat suitability for corals in areas that have not been surveyed and produced at spatial resolutions sufficient to guide research vessels towards clearly defined areas for sampling activity. The data from the resulting surveys can then be used to develop high-resolution local-scale models for that area and to verify coral presence or absence.

Coarse spatial resolutions at global and regional scales, such as those described in Davies et al. [4] and Tittensor et al. [6] are not sufficient for identifying future sampling targets and management applications (i.e. potential MPA identification). In this study, the previously published modelling approach described by Davies et al. [4] has been revised to use the highest resolution, 30-arc second, global bathymetric data available (effectively 1 km^2^ cell resolution) to resample coarse resolution global environmental datasets. The 30-arc second environmental database, presented here, generates representations of seafloor conditions, which can be highly variable over small spatial scales. The high resolution environmental datasets were then used to predict potential habitat suitability for five species of framework-forming scleractinians.

## Methods

### Coral presence data

**Figure 1:**
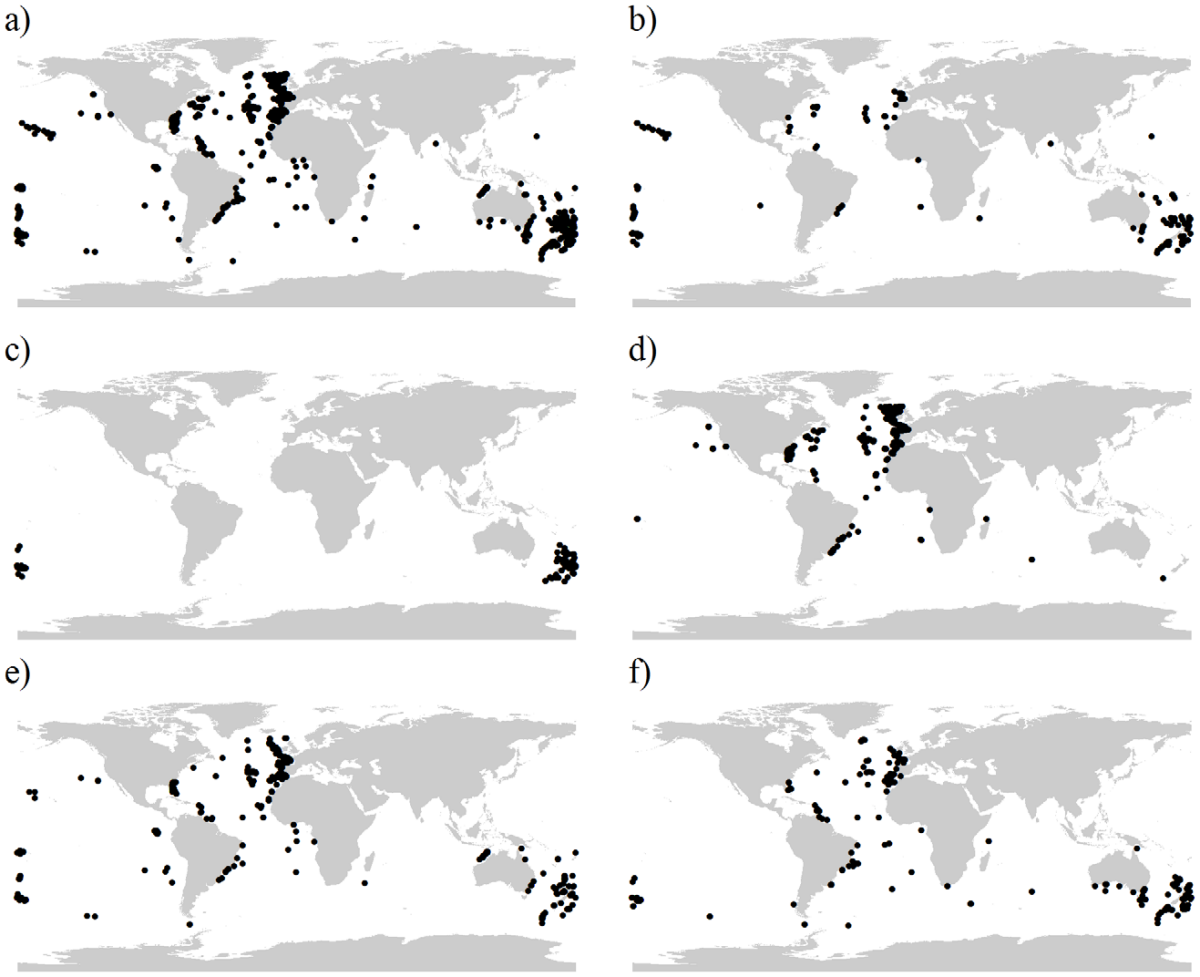
Global distribution of species presences used in this study. a) All five framework forming species, b) *Enallopsammia rostrata*, c) *Goniocorella dumosa*, d) *Lophelia pertusa*, e) *Madrepora oculata*, f) *Solenosmillia variabilis*.

The majority of species presence data for (number of presence localities retained for analysis in brackets), *Enallopsammia rostrata* (215), *Goniocorella dumosa* (230), *Lophelia pertusa* (863), *Madrepora oculata* (591) and *Solenosmillia variabilis* (380), were collated from several sources including online databases, the United Nations Environment Programme and from sources such as peer-reviewed journals, museum records, cruise reports and grey literature (See Figure 1 and Table S1). *Oculina varicosa* was omitted from analysis due to the paucity of records. In total, 2,279 presence localities were retained for individual species analysis after removing duplicate records and those records that fell outside of the analysis area. All framework forming species were combined to create an analysis of scleractinian framework-formers, 1,697 localities were retained for the combined species analysis, as multiple species were sometimes found within a single sampling location. Multiple coral locations within the same 30-arc second cell weight the habitat suitability values in favour of the environmental conditions that exist in these cells. This bias was removed by retaining only one coral presence for multi-species assemblages.

### Environmental data

Thirty two environmental layers were produced for use in the predictive models. These datasets were collated from sources that included ship CTD data, satellites (e.g. MODIS), climatologies such as World Ocean Atlas and modelled data (Table 1). The majority of source data were available as gridded datasets partitioned into bins at standardised depth levels (z-layers), and ranged in depth from 0 to ∼5500 m (z-binned datasets, e.g. temperature), whilst others were available as only a single layer at the surface (e.g. surface primary productivity) (Table 1). For z-binned datasets, it was assumed that the conditions found at a specific gridded depth were representative of conditions at that area of seafloor. This allows for the creation of continuous representations of seafloor conditions by extrapolating each z-bin to the corresponding area of seafloor at that depth using an up-scaling approach. Significant improvements over earlier methods, such as the approach of Davies et al. [4], have been achieved by integrating the highest resolution global bathymetric dataset available (SRTM30 [18], a 30-arc second dataset, approximately 1 km^2^) to allow for the preservation of a higher spatial resolution.

**Table 1:** Environmental layers developed for this study.

Variable	Native resolution	Source
*Terrain variables*		
Depth	0.0083°	Becker et al. [18]
Slope	0.25°×0.2°	Becker & Sandwell [19]
Rugosity^1^^,^ 3, slope 2^2^^,^ 3	0.0083°	Derived from Becker et al. [18]
*Hydrographic variables*		
Regional current flow, vertical flow	0.5°	Carton et al. [20] 4 ^,^ c
*Chemical variables*		
Alkalinity	3.6°×0.8–1.8°	Steinacher et al. [21] 5 ^,^ b
Apparent oxygen utilisation, dissolved oxygen, percent oxygen saturation.	1°	Garcia et al. [22] a
Aragonite and calcite saturation states	1°, 3.6°×0.8–1.8°	Orr et al. [23] 6 ^,^ a, Steinacher et al. [21] 5 ^,^ b
Carbonate ion concentration	3.6°×0.8–1.8°	Steinacher et al. [21] 5 ^,^ b
Dissolved inorganic carbon	3.6°×0.8–1.8°	Steinacher et al. [21] 5 ^,^ b
Nitrate, phosphate, silicate	1°	Garcia et al. [24] a
pH	1°, 3.6°×0.8–1.8°	Orr et al. [23] 6 ^,^ a, Steinacher et al. [21] 5 ^.^ b
Salinity, temperature	0.25°	Boyer et al. [25] a
*Biological variables*		
Particulate organic carbon	0.08°	Lutz et al. [26]
Primary productivity	0.04°	MODIS L3 Annual SMI^7^
Primary productivity export	0.05°	Behrenfield & Falkowski [27] 8

a Available in 33 z-bins ranging from 0–5500 m.

b available in 25 z-bins ranging from 6–4775 m.

c available in 40 z-bins ranging from 5–5374 m.

1 Derived using Bathymetric Terrain Modeler.

2 Derived using ArcGIS spatial analyst.

3 4 layers created using moving windows of 5 km, 20 km, 30 km and 100 km.

4 SODA model 2.0.4; mean 1990–2007.

5 Extracted from SRES B1 scenario model; mean 2000–2009.

6 Extracted from OCMIP2 model data for 1995.

7 Downloaded from http://oceancolor.gsfc.nasa.gov, MODIS L3 product; mean 2002–2008.

8 Standard VGPM using MODIS data; mean 2002–2007.

Converting the z-binned datasets into representations of seafloor conditions involved several processes. Firstly, for each variable, all available z-bins were extracted independently and interpolated to a slightly higher spatial resolution (usually 0.1°) using inverse distance weighting. This interpolation procedure was required to minimise gaps that appeared between adjacent z-bins due to non-overlap when extrapolated on to the bathymetry. Secondly, these layers were then resampled to match the extent and cell resolution of the bathymetry with no further interpolation. Thirdly, each resampled z-bin was then draped over the area of seafloor that corresponded to its depth range. Each of these bins did not overlap, and were finally merged to produce a continuous representation of the variable at the seafloor. It was assumed that conditions beyond the maximum depth of the data used in this study (>5500 m) were relatively stable towards the maximum depth of the area. This was unlikely to influence suitability models as framework-forming cold-water corals have not been documented at these depths. This method was used to create annual-mean values for regional current velocity, temperature, salinity, nitrate, phosphate, silicate, dissolved oxygen concentrations and carbonate chemistry parameters (Table 1).

Surface datasets were not up-scaled by the above process as they were only available as a single z-bin. Instead, these variables were initially interpolated to a higher spatial resolution (usually 0.1°) using inverse distance weighting and then resampled to match the extent and cell resolution of the other variables. Surface productivity values were obtained from the Vertically Generalised Productivity Model (VGPM, [27]) and MODIS chlorophyll *a* data (years 2002–2007), and particulate organic carbon flux to the seafloor was obtained from Lutz et al. [26]. Terrain variables were calculated using ESRI's ArcGIS 9.3 Spatial Analyst extension or the Benthic Terrain Modeler (BTM) extension [28] using SRTM30 bathymetry. Bathymetric position index and rugosity were calculated using BTM. Slope and aspect were calculated within ArcGIS and converted to continuous radians. Additional slope data were obtained from analyses by Becker & Sandwell [19] for comparison with slope computed in this study. Temporal variability within variables was omitted, as the longevity of many cold-water coral species far exceeds the measuring period of most oceanographic variables. For example, in regions not impacted by recent glacial activity, there is evidence for continuous cold-water coral growth over the last 50,000 years [29].

**Figure 2:**
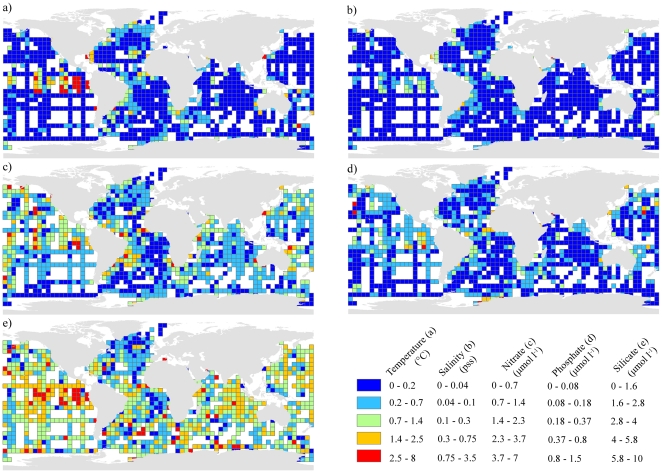
Geographical distribution of error between the up-scaled environmental layers and water bottle stations. a) Temperature, b) salinity, c) nitrate, d) phosphate, e) silicate. The scales show the average difference of all water bottle stations that fall within a single five degree grid cell.

The accuracy of the up-scaled environmental variables was tested using quality controlled water bottle data obtained from the Global Ocean Data Analysis Project (GLODAP version 1.1; [30]). GLODAP data was available for temperature, salinity, nitrate, phosphate and silicate. GLODAP values deeper than 50 m were retained for analysis and validation was conducted by intersecting the location of GLODAP stations with the corresponding 30-arc second environmental layers. Relationships were statistically analysed using Pearson's correlation. To show the spatial distribution of error throughout the world's oceans, the average difference between GLODAP stations and the up-scaled environmental layers were plotted onto a five degree grid (Figure 2).

### Predicting distribution

The Maxent modelling (maximum entropy modelling [31]) approach was chosen to: (1) collectively model all scleractinian framework forming coral species (5 species, omitting *O. varicosa* due to a paucity of records) for management applications (i.e. identifying potential VMEs) and (2) model five species of reef forming scleractinians (*E. rostrata*, *G. dumosa*, *L. pertusa*, *M. oculata* and *S. variabilis*) individually. Maxent is a presence-only approach that generally out performs other presence-only techniques including Ecological Niche Factor Analysis (ENFA) [6], [32]. The underlying assumption of Maxent is that the best approach to determining an unknown probability distribution (in this case, the distribution of a cold-water coral species) is to maximise entropy based on constraints derived from environmental variables [31]. The algorithm is supplied within a Java software package (Maxent version 3.2.1). The default model parameters were used as they have performed well in other studies (a convergent threshold of 10^−5^, maximum iteration value of 500 and a regularisation multiplier of 1, [33]).

Covariation between environmental datasets is a complication that must be addressed in many predictive modelling efforts. Environmental datasets used in this analysis were assessed for covariation in a correlation matrix (Figures S1 and S2). Although Maxent is reasonably robust with respect to covariation, an *a priori* variable selection process was used to reduce covariation. Variables were selected based on a literature search of environmental factors known or thought to influence cold-water coral growth and survival. Strong correlations between variables (>0.7) were addressed by omitting one of the environmental variables (except for aragonite saturation state and temperature; see results and discussion). The importance of each variable in the model was assessed using a jack-knifing procedure that compared the contribution of each variable (when absent from the model) with a second model that included the variable. The final habitat suitability maps were produced by applying the calculated models to all cells in the study region, using a logistic link function to yield a habitat suitability index (HSI) between zero and one [33].

Several studies have highlighted issues with using only one statistic to evaluate model performance (see [34]). In this study, the model accuracy between the test data and the predicted suitability models was assessed using a threshold-independent procedure that used a receiver operating characteristic (ROC) curve with area under curve (AUC) for the test localities and a threshold-dependent procedure that assessed misclassification rate. To calculate validation metrics, the presence data was randomly partitioned to create 70% training and 30% test datasets, with test data used to calculate validation metrics. With presence-only data, Phillips et al. [31] define the AUC statistic as the probability that a presence site is ranked above a random background site. In this situation, AUC scores of 0.5 indicate that the discrimination of the model is no better than random and the maximum achievable AUC value is 1. Several studies have criticised the use of AUC as a single metric for assessing performance because AUC is sensitive to the total spatial extent of the model [35], [36]. In this study, the presence localities of some coral species were restricted to isolated regions (i.e. most *G. dumosa* records are located in the waters surrounding New Zealand), in these cases, AUC scores may be inaccurate. Two further metrics were applied, 1) a threshold-dependent omission rate (fixed value of 10) [37], which evaluates model success by assessing the proportion of test locations that fall into cells that were not predicted as suitable, and 2) Test gain, which can be interpreted as the average log probability of the presence samples used to test the model. For example, if the test gain is 2, the average likelihood of a test presence locality is exp(2) (about 7.4) times greater than that of a random background pixel [38].

There is ongoing debate regarding the interpretation of Maxent's logistic prediction values (0–1) for habitat suitability [35], [39]. Rather than assign an arbitrary cut-off, several studies have defined a binary threshold, which states that a species is likely to be found in an area with a habitat suitability value above a given threshold, but not likely to be found below it [37], [40], [41]. Maxent's 10^th^ percentile (presence value) was used to provide a cut-off point for suitability in this study. The assumption being that 10% of the presence data may occur in areas where the species is absent due to positioning errors or a lack of resolution in environmental data, and as such, omits the suitability values below the highest of the 10% of records. This is especially pertinent for the coral species locations presented here, as presence records were collected over long time periods with varying degrees of accuracy in spatial precision [37], [41].

## Results

### Environmental layers

**Figure 3:**
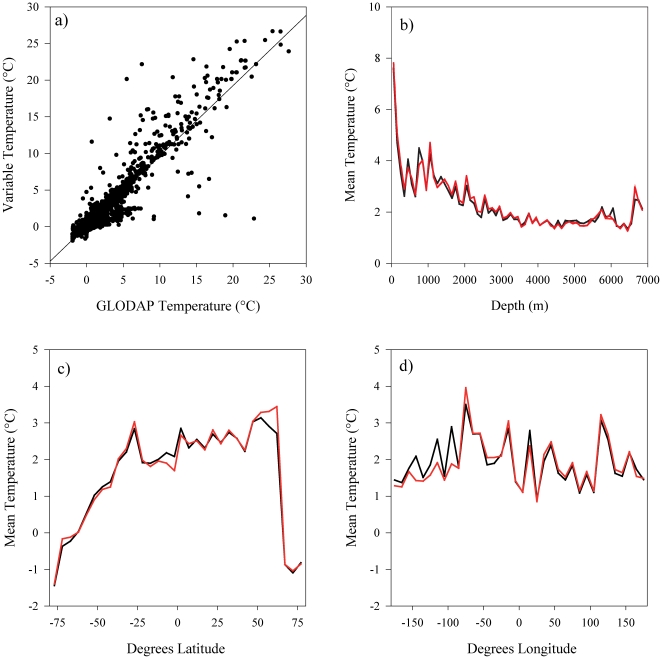
Validation of the environmental layer creation process for temperature. a) Correlation (0.924) of intersected GLODAP stations with the layer, b) mean temperature relationships at depth in 50 m bins, c) mean temperature at latitude in 5° bins, d) mean temperature at longitude in 10° bins. The black lines are temperature at each GLODAP bottle station; the red lines are the value of the environmental layer at the position of each GLODAP station.

The five up-scaled environmental variables that were assessed with GLODAP water bottle data were highly correlated at each sampling location (Pearson's correlation, R^2^, temperature = 0.924 (*n* = 6972), salinity = 0.914 (*n* = 6891), nitrate = 0.913 (*n* = 6598), phosphate = 0.923 (*n* = 6386), silicate = 0.823 (*n* = 6994), all values significant at *p*<0.001) (Figures 3 and Figure S3, Figure S4, Figure S5, Figure S6). Temperature correlated the strongest with GLODAP data (Figure 3a) and generally reflected the patterns observed in the GLODAP data (Figure 3b). Similar patterns between the validation data and the environmental layer were observed along latitudinal gradients with a slight mismatch south of the equator and between 50° and 60°N (Figure 3c). Longitudinally, the layer underperformed between 80° and 180°W, but performance increased eastward (Figure 3d). Shown spatially, the discrepancies between the variables and water bottle data are generally found in areas of high variability, i.e. in the Pacific Ocean and/or areas where upwelling occurs (Figure 2). The other four variables showed similar patterns as temperature, with consistent fit across depth, longitudinal and latitudinal gradients (Figure S3, Figure S4, Figure S5, Figure S6). Variables that were up-scaled using source data with higher native spatial resolutions (i.e. temperature and salinity, 0.25°) performed better than variables with native resolutions of 1° (i.e. silicate, phosphate and nitrate) (Figure 2), but the response was not consistent amongst these variables with silicate showing more spatial variability than nitrate and phosphate (Figure 2c–d).

### Species niches

From the suite of environmental variables available, the *a priori* variable selection identified eight variables that were likely to influence the probability of species presence (Table 2). Two variables that were highly correlated, but were retained on the strength of their contribution were aragonite saturation state (Ω_ARAG_) and temperature, which were positively correlated for both species and randomly distributed points (0.89 and 0.83 respectively, Figures S1 and S2). The jack-knife analysis of variable contribution showed that amongst the scleractinian species the highest contributions were from temperature, Ω_ARAG_, depth and salinity. This must be interpreted with caution due to covariation as these layers can contain similar information, which may artificially inflate variable contribution scores. However, the test AUC scores for models generated with a single variable reinforced that these variables were the top predictor variables regardless of covariation.

**Table 2:** Validation statistics and jack-knife analysis of variable contributions to the models.

Variable	All 5 species	*E. rostrata*	*G. dumosa*	*L. pertusa*	*M. oculata*	*S. variabilis*
*Validation statistics*						
Test AUC	0.986 (0.002)	0.971 (0.015)	0.996 (0.001)	0.993 (0.002)	0.990 (0.002)	0.985 (0.004)
Test gain	3.082	3.393	4.873	4.150	3.621	3.351
10th percentile training presence	0.590	0.392	0.678	0.678	0.538	0.464
Omission rate (Threshold 10)	2%	7.8%	4.4%	0.8%	1.7%	3.5%
*Jack-knife of variable importance*						
Depth	**1.506**	**2.098** †	**2.880** †	1.986	**2.015** * †	**1.980** †
Dissolved oxygen	0.101	0.173	0.751*	0.343	0.100	0.257
Aragonite saturation state (Ω_ARAG_)	**1.445**	**1.761**	**2.805**	**2.094**	**1.876**	**1.809**
Particulate organic carbon	1.177	1.141*	2.507	1.720	1.422	1.571
Phosphate	0.832	0.583	1.702	1.683	1.127	0.554
Salinity	1.272	1.673	1.747	**2.208** * †	1.681	1.680
Slope (100 km)	0.317	0.718	0.167	0.381	0.615	0.622
Temperature	**1.508** * †	**2.007**	**2.845**	**2.168**	**1.965**	**1.710** *
*Test AUC for a single variable*						
Depth	**0.966**	**0.954**	**0.984**	0.966	**0.972**	**0.953**
Dissolved oxygen	0.665	0.744	0.838	0.705	0.715	0.784
Aragonite saturation state (Ω_ARAG_)	**0.964**	0.917	**0.983**	**0.981**	**0.968**	**0.958**
Particulate organic carbon	0.941	0.914	0.972	0.963	0.942	0.937
Phosphate	0.889	0.797	0.914	0.964	0.891	0.809
Salinity	0.963	**0.927**	0.950	**0.984**	0.941	**0.963**
Slope (100 km)	0.758	0.825	0.656	0.763	0.812	0.764
Temperature	**0.970**	**0.946**	**0.984**	**0.984**	**0.974**	0.952

Higher values for the regularised training gain of the jack-knife test indicates greater contribution to the model for a variable (these values are not directly comparable between the different species). Test AUC numbers in parentheses are the standard deviation of the Test AUC scores.

*indicates the variable that reduced the gain the most when omitted and therefore contained the most information that was not present in other variables.

†indicates the variable with the highest gain when used in isolation and had the most useful information by itself. The top 3 variables are highlighted in bold for each species, both for jack-knife of variable contribution and test AUC values for Maxent models generated using a single variable.

**Figure 4:**
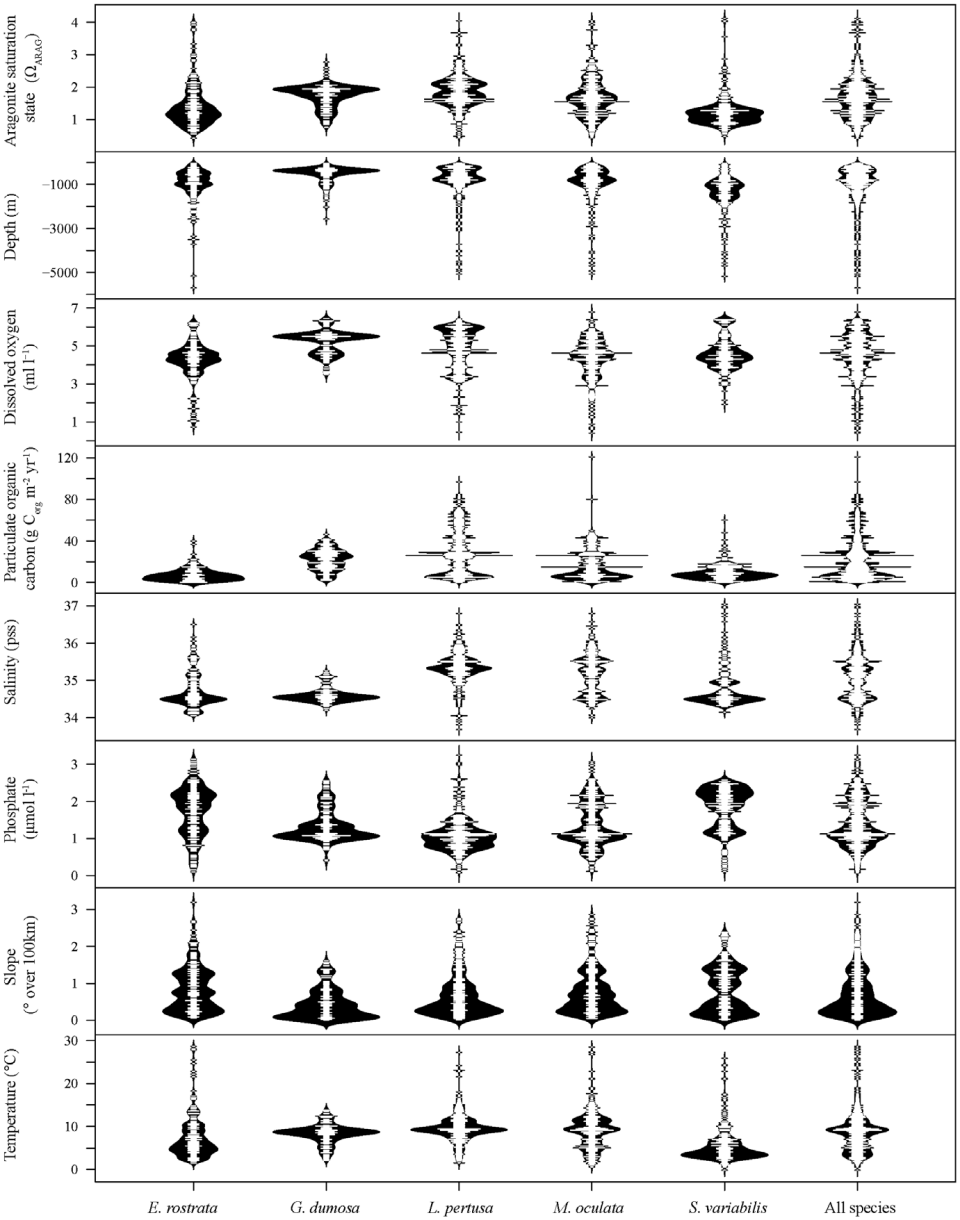
Bean plots of species presences intersected with the environmental variables used in the models (the small lines in the centre of each bean shows individual presence data points. The bean itself is a density trace that is mirrored to show as a full bean [42]).

By intersecting the known distribution of coral species with the environmental layers, it was possible to gain insight into the species niches and the factors that are most important in controlling their distribution (Figure 4 and Table S2). For Ω_ARAG_, most coral records were found in waters supersaturated with respect to aragonite (Ω_ARAG_>1; 88.5% of all records). Most species were restricted to depths shallower than 1500 m, but there were some records (11%) that were found much deeper and are likely to be errors in the reporting of the species' position, especially on seamounts or steep slopes. The majority of coral records were found in areas where dissolved oxygen concentrations were >4 ml l^−1^. *Enallopsammia rostrata* and *S. variabilis* were mostly found in areas with limited particulate organic carbon input. However, *G. dumosa*, *L. pertusa* and *M. oculata* occur across a greater range of productivity, between 5 and 120 g C_org_ m^−2^ yr^−1^. All species in this study had a relatively limited salinity range between 34 and 37 (Figure 4 and Table S2). *Goniocorella dumosa*, *L. pertusa* and *S. variabilis* were found in areas with restricted temperatures of ∼8°C, 10°C and 5°C respectively, whilst *E. rostrata* and *M. oculata* were found over wider temperature ranges (Figure 4 and Table S2). In general, scleractinian framework-forming corals were mostly found in areas (but are not limited to) that are: 1) supersaturated with respect to aragonite, 2) <1500 m in depth, 3) with dissolved oxygen concentrations >4 ml l^−1^, 4) over a relatively limited salinity range, 5) with low nutrient concentrations and 6) temperatures between 5–10°C (Figure 4 and Table S2).

### Model evaluation

The coral habitat models generated performed well across the metrics used to validate the modelled outputs. All AUC scores were >0.97 (Table 2) and were significantly different from that of a random prediction of AUC = 0.5 (Wilcoxon rank-sum test, *p*<0.01). The AUC score for the scleractinian habitat model that included all species performed better than some individual species models, which suggests the niches of individual scleractinian species had some overlap for the most important variables (see species niches subsection above and Table 2). The high AUC scores were supported by high test gain and low omission rates across the species, indicating only few presences were misclassified as absent and that predicted presences were several orders of magnitude more probable than that of a random background pixel (Table 2).

### Habitat suitability

**Figure 5:**
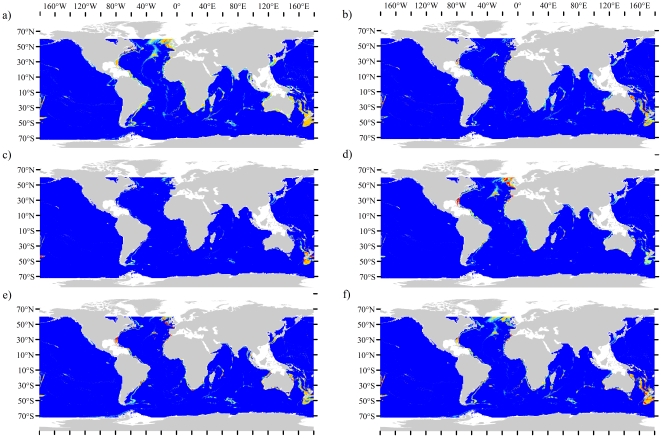
Habitat suitability maps for framework forming species. a) All five framework forming species, b) *E. rostrata*, c) *G. dumosa*, d) *L. pertusa*, e) *M. oculata*, f) *S. variabilis*. High resolution maps are available as supplementary Figures S7, S8, S9, S10, S11, S12.

**Figure 6:**
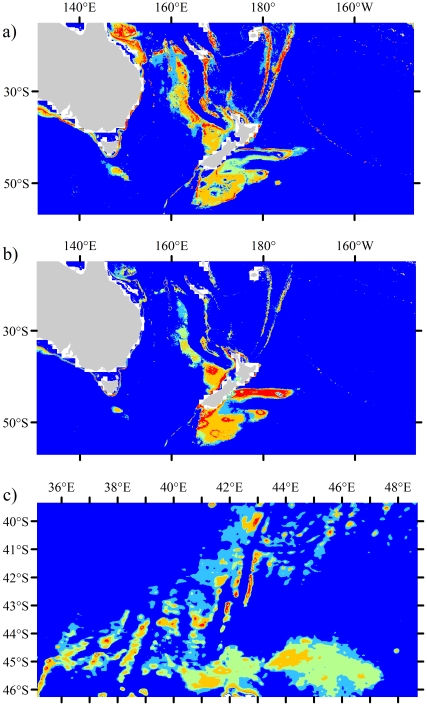
Local and regional area outputs for areas suitable habitat. a) *E. rostrata* around New Zealand and Australia, b) *G. dumosa* around New Zealand and Australia, c) *S. variabilis* on seamounts in the Southern Ocean south of Madagascar.

**Figure 7:**
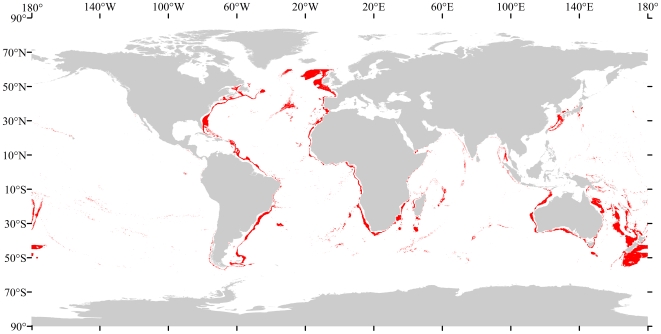
Binary predicted presence map for scleractinian framework-forming corals based on the 10^th^ percentile training presence, which omits the 10% most extreme presence observations as they may represent recording errors. White background indicates that these species are not likely to be found, red indicates probable presence.

The benefit of higher resolution environmental layers is immediately obvious in the habitat suitability maps generated by Maxent (Figure 5). Maxent identified suitable habitat for framework-forming cold-water corals throughout the world's oceans (Figure 5a), but individual species habitat suitability varied greatly by geographic region (Figures 5b–5f, 6 and Figure S7, Figure S8, Figure S9, Figure S10, Figure S11, Figure S12). The majority of suitable habitat was predicted in the North Atlantic and the South Pacific (waters surrounding New Zealand). Other significant regions for scleractinian habitat included the continental shelves off Western Africa, Eastern South America and Western/Southern Australia. Predicted coral habitat in the Indian Ocean, Central Pacific, and Southern Atlantic was largely limited to large seamounts (>1 km in diameter) and deep slopes of oceanic islands. On a global scale, both continental margins and seamounts are known to be important areas for deep-sea scleractinian reef-formers (Figures 5, 7 and S7). However, there are likely many smaller seamounts (<1 km in diameter) that were not detected in the models.

The model outputs showed distinct geographic separation in the distribution of suitable habitat for each species (Figures 5b–5f, 6 and Figure S7, Figure S8, Figure S9, Figure S10, Figure S11, Figure S12). *Enallopsammia rostrata* was largely predicted to be found in the South Pacific (Figures 1b, 5b, 6a and S8). The majority of suitable habitat for *G. dumosa* was predicted in the waters of New Zealand and Australia. Suitable habitat for *G. dumosa* was also found on the continental shelves of Northern Europe, but this should be interpreted with caution due to the limited sampling distribution for this species. In contrast to *E. rostrata*, *G. dumosa* was less prevalent on large seamounts (Figures 5c, 6b and S9). Suitable habitat for *L. pertusa* was largely restricted to the North East Atlantic and the South Eastern USA (Figures 5d and Figure S10). The majority of *L. pertusa* habitat was predicted on continental shelves and slopes, with less suitable habitat predicted on seamounts. *Lophelia pertusa* habitat was also predicted in the waters of New Zealand, an area where living colonies have not been documented. The distribution of suitable habitat for *M. oculata* was similar to *L. pertusa*, but was more prevalent on large seamounts (Figures 5e and Figure S11). Finally, suitable habitat for *S. variabilis* appears throughout the world's oceans, but was largely restricted to large seamounts and in the waters surrounding New Zealand. Suitable *S. variabilis* habitat was also predicted on the continental slopes of the Atlantic Ocean and throughout the Mid-Atlantic Ridge (Figure 5f, 6c and S12). High resolution images of habitat suitability by species are available as supplementary figures (Figure S7, Figure S8, Figure S9, Figure S10, Figure S11, Figure S12).

## Discussion

This study improves significantly on previous global and regional modelling efforts such as those by Davies et al. [4] and Tittensor et al. [6]. The up-scaling approach used to characterise seafloor conditions at 30-arc second spatial resolution resulted in a global database of environmental, chemical and physical variables, which could be used to predict the distributions of non-coral deep-sea taxa. The increase in spatial resolution resulted in significantly more presence records being included in the models than in previous studies [4]. However, despite the advantages of this approach there are still several limitations and constraints that must be recognised in modelling deep-sea organisms at global scales (see below).

### The positives and negatives of up-scaling

Issues of spatial accuracy and scale have frustrated ecologists and modellers for decades. In particular, the selection of appropriate spatial and temporal resolutions for environmental datasets is an important factor when constructing habitat suitability models [43]. In previous studies, determination of relevant spatial resolutions was difficult and/or unattainable for cold-water corals (i.e. 1° in Davies et al. [4] and Tittensor et al. [6]). Coarse resolution models miss important bathymetric features such as seamounts and canyons, which are known to harbour well developed cold-water scleractinian ecosystems. Depth can vary considerably over small spatial scales and these undersea features must be captured in modelling efforts. This is particularly noticeable in areas that have strong environmental gradients over short distances such as where two water masses meet and create a clearly defined front over a distance of only several hundred metres (i.e. temperature in the Faeroe Shetland channel [4], [44]).

Whilst there are numerous benefits to the high resolution of the up-scaling approach, there are still several issues that must be considered. Firstly, the success of the environmental up-scaling approach is heavily dependent on the quality and native resolution of the input data. Up-scaled variables with higher native resolutions had greater agreement with water bottle data than those at coarser resolutions (0.25° for temperature and salinity against 1° for nitrate, phosphate and silicate) (Figure 3). Secondly, global climatologies such as World Ocean Atlas produce annual averages that bin all available data from multiple time series into a single data product to retain a higher number of samples and hence greater spatial coverage. Monthly or seasonal time series are often made available, but suffer from reduced sample numbers that increases the uncertainty in the data. Thirdly, the reinterpolation of the source data which comprises a component of the variable up-scaling process also introduces error. This produces a smoother response in some areas (Figures 2, S1, S2, S3, S4) and is most noticeable between 100° and 180°W, but the general pattern between the up-scaled variables and the GLODAP test data was similar. Fourthly, the up-scaling procedure generalises conditions for a given area of seafloor and did not incorporate small scale oceanographic variability such as upwelling or downwelling on seamounts or banks, which is probably not captured in source data with low native resolutions (Figure 3).

**Figure 8:**
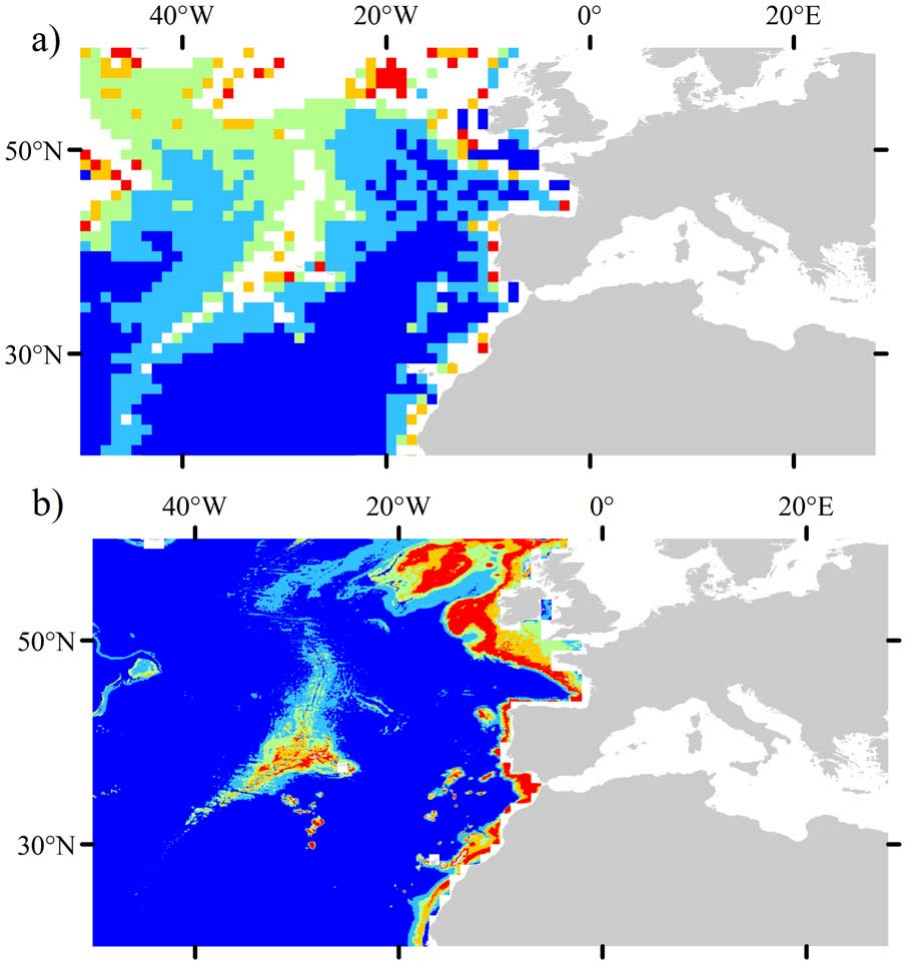
Comparison between earlier predictions of suitable habitat for *L. pertusa* by Davies et al. [**4**] and those developed in this study. a) global *L. pertusa* habitat predicted by Davies et al. [4] using ENFA, b) global *L. pertusa* habitat predicted by this study. Note the significant increase in spatial resolution and ability to identify suitable habitat on seamounts off Portugal.

There are some areas where the up-scaled environmental layers are less reliable for a combination of the reasons listed. For example, there are lower numbers of observations in source datasets between 100° and 180°W compared with well studied regions such as the North Atlantic [24], which leads to some discrepancies between the up-scaled layers and water bottle data (Figure 3). Some regions also contain large scale oceanographic features that vary temporally, for example, the up-scaled temperature layer showed large inconsistencies in the area of the El Niño/La Niña-Southern Oscillation (central Pacific), which was captured in bottle data. In general, most variables with the exception of silicate performed well in the Atlantic, Indian and Southern Oceans (Figure 3). These points highlight the problem with uneven sampling effort throughout the world's oceans, the coarse native resolutions and the coarse temporal resolutions at which data are available. On the whole, these minor errors do not distract from the capability of the up-scaling approach to produce fairly accurate representations of conditions on the seafloor (Figure 8), but care must be taken when interpreting the modelled habitat suitability in areas where the environmental data may be less reliable (Figure 3).

### Unincorporated and limited geographic extent of model variables

There are several variables that are important for scleractinian coral settlement, growth and survival that were not included in the model because they do not exist at sufficient resolutions and/or at global scales. These variables include benthic hard substrata, high resolution current direction/velocity and mobile or benthic sediments. Framework-forming scleractinians require hard substrata for colonisation (e.g. *L. pertusa* [45]) and like depth, substrate tends to be highly variable over small spatial scales. Vast areas of hard substrate may not be required in all areas, as small cobbles and shells may represent attachment substrata in the early stages of reef development [45] but this often depends on environmental requirements being met in the region and that sufficient larval supply is present. Similarly, current velocity and direction also vary considerably over small spatial scales [46]. For example, on the Jasper Seamount in the Pacific, octocorals are more abundant near peaks and on small-scale topography such as knobs and pinnacles compared with mid-slope sites at similar depths [47]. It is likely that this is also true of scleractinian corals on seamounts, as previous observations amongst reefs have shown them to be largely found on undersea features where encounters with food particles are maximised [46], [48]. Cold-water scleractinian corals also appear to be adversely affected by heavy sedimentation and consequently areas with high sediment loads and soft bottoms may not be suitable for coral colonisation or survival [49]. For local and regional scale modelling, it is important that substrate, current velocity/direction and sediment data be included when available. Recent work on developing proxies for substrate shows great promise in areas where multibeam bathymetry or side-scan sonar has been collected [50].

Model results presented here likely overpredict the amount of suitable habitat in some areas because fine-scale bathymetric features (10's of metres), substrate and current data are not available. These overpredictions were especially evident in the North East Atlantic and the South East USA (Figure 5). Both areas are known to support well developed cold-water coral ecosystems [51], but the model results indicate suitable coral habitat in areas that are known soft bottom regions where corals are likely or known to be absent. Over-prediction could also be a problem in other coastal regions that have high sediment loadings (i.e. the east coast of South America) and/or the presence of soft substrata.

In addition to several unincorporated datasets, the geographic extent of some important variables (i.e. Ω_ARAG_) was limited and reduced the extent of the model analysis. In this study, present day carbonate chemistry data from Orr et al. [23] was selected over Steinacher et al. [21] because it was based on modern-day observations from survey data [52], used a multi-model approach, was available at a higher spatial resolution (1° versus 3.6°×0.8–1.8°) and was modelled on more z-bins (33 versus 25). The disadvantage of using Orr et al. [23] over Steinacher et al. [21] is that the analysis extent was limited to a maximum of 60°N and omitted the Gulf of Mexico, South China Sea and the Mediterranean Sea. The restriction at 60°N omitted some of the best developed and documented *L. pertusa* reefs in the north Atlantic [53]. The two Ω_ARAG_ datasets were a reasonable fit at the locations where scleractinian records were found (Pearson's correlation, R^2^ = 0.85, *n* = 2,279, *p*<0.001), but there were large differences in the proportion of species records that were found in waters undersaturated with aragonite (11.5% were found in undersaturated waters in Orr et al. [23] and 5.4% in Steinacher et al. [21]). These differences were more pronounced amongst the deeper species in this study, i.e. for *E. rostrata* and *S. variabilis*, 25.1% and 30.3% of records respectively were found to be undersaturated in Orr et al. [23] compared with 11.2% and 11.1% in Steinacher et al. [21]. These differences arise mostly from the greater vertical and cell resolution of Orr et al. [23], which produces better fitted environmental variables using the up-scaling approach presented in this manuscript. The Steinacher et al. [21] data extends into the Arctic (>60°N) but is derived from limited modern-day observations, which are needed to accurately model carbonate chemistry in the region. The extent, quality, and availability of environmental, chemical and physical data are continually improving and should be incorporated in an iterative process with field surveys to refine predictions and reduce the number of false positives and negatives in habitat suitability models.

### Presence records and variable importance

The limited number of coral presence records used to model habitat distribution for some species highlights the need for more targeted sampling to document coral locations globally. For example, few *O. varicosa* presence localities were obtained and preliminary models suffered from significant overprediction and artificially high AUC scores, forcing the omission of this species from the analysis. Several recent studies have investigated the effectiveness and reliability of habitat suitability models constructed with low numbers of presences, a common problem for difficult to detect species (i.e. cold-water corals) and those that have had limited systematic survey effort such as records from museum collections [54]. This does not preclude the possibility of modelling species distributions with low sample numbers, as Maxent is capable of producing good models with as few as five presences [37]. However, Maxent does appear to overpredict suitable habitat when using small presence datasets compared with other methods [37], [55]. In this study, the amount of presence records for *E. rostrata* and *G. dumosa* were comparatively lower than the other species, but this study has used more presence records than previous global deep-sea habitat suitability models [4], [6].

Depth, temperature, salinity and aragonite saturation state accounted for the highest contributions to coral habitat predictions and agree with findings from previous studies into cold-water coral distributions [4], [6], [56]. Particulate organic carbon (POC) was expected to be an important variable as cold-water corals are sessile filter feeders dependent on organic matter falling from the surface or advected via currents that bring organic matter and zooplankton to the coral [57]. The majority of coral records retained in this analysis were located in areas with relatively low POC flux, which suggests several hypotheses. 1) That cold-water scleractinians may not be as dependent on high surface productivity as suggested by Guinotte et al. [14], as food may be transported into coral areas from adjacent waters with higher productivity. 2) The cold-water species included in this analysis have relatively low nutritional requirements or 3) the input data does not accurately capture the POC reaching the seafloor. Further research into the nutritional requirements of cold-water scleractinians is required to satisfy these hypotheses. Additionally, the proportion of records of *E. rostrata* and *S. variabilis* found in areas undersaturated with aragonite were much greater, 25.1% and 30.3% respectively, compared to the other three species included in the analysis (*G. dumosa* 4.4%, *L. pertusa* 2.7% and *M. oculata* 10.3%). This suggests that *E. rostrata* and *S. variabilis* are potentially less susceptible to the shoaling of the aragonite saturation horizon than other framework-forming scleractinians as they are found in deeper waters that are already closer to the aragonite saturation horizon. This fact highlights the paucity of information available on how cold-water corals may respond to changes in basic environmental conditions and supports the need for further, multi-species, experimental investigation into their tolerances.

### Field validation and utility of habitat predictions for management

Field validation of modelled habitat is needed to 1) assess the accuracy of model predictions, 2) refine models by identifying false positives, and 3) gauge the utility of these methods for identifying cold-water coral habitat in unsurveyed areas for management action (i.e. the high seas). The model results presented here are not meant to identify coral occurrences with pin point accuracy and are unlikely to achieve this based on currently available data. They are more useful in directing research effort to areas that have the highest probability of supporting framework-forming cold-water corals. One additional complication for field validation efforts using these high resolution predictions are the current technological limitations of survey vehicles and equipment (i.e. ROVs, submersibles, drop cameras, etc). The distribution of cold-water coral ecosystems within a single cell of these models (30-arc seconds) could be patchy [45] and could easily be missed on vehicle transects with limited range and narrow fields of view. To address this limitation, and to improve the probability of locating undiscovered coral areas, research ships should first use multibeam surveys (in high probability areas) to identify substrate characteristics that can support framework-forming cold-water coral growth or identify corals (e.g. emergent hard substrata, coral rubble). These substrates have distinct acoustic backscatter signatures in multibeam bathymetry and can be used to target the deployment of video cameras or ROVs which may reveal cold-water coral ecosystems [50], [58].

### Conclusions

The high costs associated with sampling and surveying in the deep sea virtually assures that detailed surveys of all of the world's oceans will not be economically feasible. This limitation highlights the need for well developed and accurate modelling efforts to identify favourable cold-water coral habitat and other vulnerable marine ecosystems such as hexactinellid sponge reefs. The up-scaling approach presented here resulted in a high resolution database of global seafloor conditions that could be used to model habitats for numerous deep-sea species. The habitat predictions and database are a significant enhancement over earlier research [4], [6], and illustrates the potential for improving our knowledge of potential cold-water coral distributions and the factors that control their distribution using existing data. Field validation of these models will increase model accuracy and future model iterations will integrate new and/or higher resolution environmental data as it becomes available. Validated models are needed to identify and document areas that should be considered for MPA designation. Regional and local scale modelling efforts in areas where higher resolution bathymetry exists (i.e. the U.S. and Australian continental shelves) will reduce overprediction, resulting in more accurate predictions of cold-water coral distribution. Regional scale models for predicting cold-water coral habitat at higher resolutions (∼90 m) are currently in development for the southeast and west coasts of the USA and represent the next step in developing predictive modelling as a valuable technique for the management of deep-sea species.

## Supporting Information

Figure S1 Correlation matrix of the environmental layers developed for this study based on 10,000 randomly distributed points throughout maximum extents (all values significant at *p*<0.05, Pearson's correlation coefficient). Colour represents correlation strength; no colour = 0–0.2, green = 0.2–0.4, yellow = 0.4–0.6, orange = 0.6–0.8 and red = 0.8–1. The negative sign in a cell represents a negative correlation between the variables, no sign denotes positive.
(TIF)

Figure S2 Correlation matrix of the environmental layers developed for this study based upon the species location data (all values significant at *p*<0.05, Pearson's correlation coefficient). Colour represents correlation strength; no colour = 0–0.2, green = 0.2–0.4, yellow = 0.4–0.6, orange = 0.6–0.8 and red = 0.8–1. The negative sign in a cell represents a negative correlation between the variables, no sign denotes positive.
(TIF)

Figure S3 Validation of the environmental layer creation process for nitrate. a) Correlation (0.913) of intersected GLODAP stations with the layer, b) mean nitrate relationships at depth in 50 m bins, c) mean nitrate at latitude in 5° bins and d) mean nitrate at longitude in 10° bins. The black lines are nitrate at each GLODAP bottle station; the red lines are the value of the environmental layer at the position of each GLODAP station.
(TIF)

Figure S4 Validation of the environmental layer creation process for phosphate. a) Correlation (0.923) of intersected GLODAP stations with the layer, b) mean phosphate relationships at depth in 50 m bins, c) mean phosphate at latitude in 5° bins and d) mean phosphate at longitude in 10° bins. The black lines are phosphate at each GLODAP bottle station; the red lines are the value of the environmental layer at the position of each GLODAP station.
(TIF)

Figure S5 Validation of the environmental layer creation process for salinity. a) Correlation (0.914) of intersected GLODAP stations with the layer, b) mean salinity relationships at depth in 50 m bins, c) mean salinity at latitude in 5° bins and d) mean salinity at longitude in 10° bins. The black lines are salinity at each GLODAP bottle station; the red lines are the value of the environmental layer at the position of each GLODAP station.
(TIF)

Figure S6 Validation of the environmental layer creation process for silicate. a) Correlation (0.823) of intersected GLODAP stations with the layer, b) mean silicate relationships at depth in 50 m bins, c) mean silicate at latitude in 5° bins and d) mean silicate at longitude in 10° bins. The black lines are silicate at each GLODAP bottle station; the red lines are the value of the environmental layer at the position of each GLODAP station.
(TIF)

Figure S7 High resolution habitat suitability map for all species.
(TIF)

Figure S8 High resolution habitat suitability map for *Enallopsammia rostrata*.
(TIF)

Figure S9 High resolution habitat suitability map for *Goniocorella dumosa*.
(TIF)

Figure S10 High resolution habitat suitability map for *Lophelia pertusa*.
(TIF)

Figure S11 High resolution habitat suitability map for *Madrepora oculata*.
(TIF)

Figure S12 High resolution habitat suitability map for *Solenosmilia variabilis*.
(TIF)

Table S1 Sources of species locality records that were utilised to develop the presence only dataset for this study. Several datasets contained identical records, which were removed, significantly lowering the number of presences available. Further records removed were, those that fell outside the analysis extent and duplicate records that fell inside a single grid cell.
(DOCX)

Table S2 Mean values of the cells of each environmental variable used in the models where species presences were found (standard deviation in parentheses).
(DOCX)
