# Supplementary figures and images for: Global Habitat Suitability for Framework-Forming Cold-Water Corals
Source: PLoS One. 2011 Apr 15;6(4):e18483. doi: 10.1371/journal.pone.0018483 (PMC3078123; doi:10.1371/journal.pone.0018483)

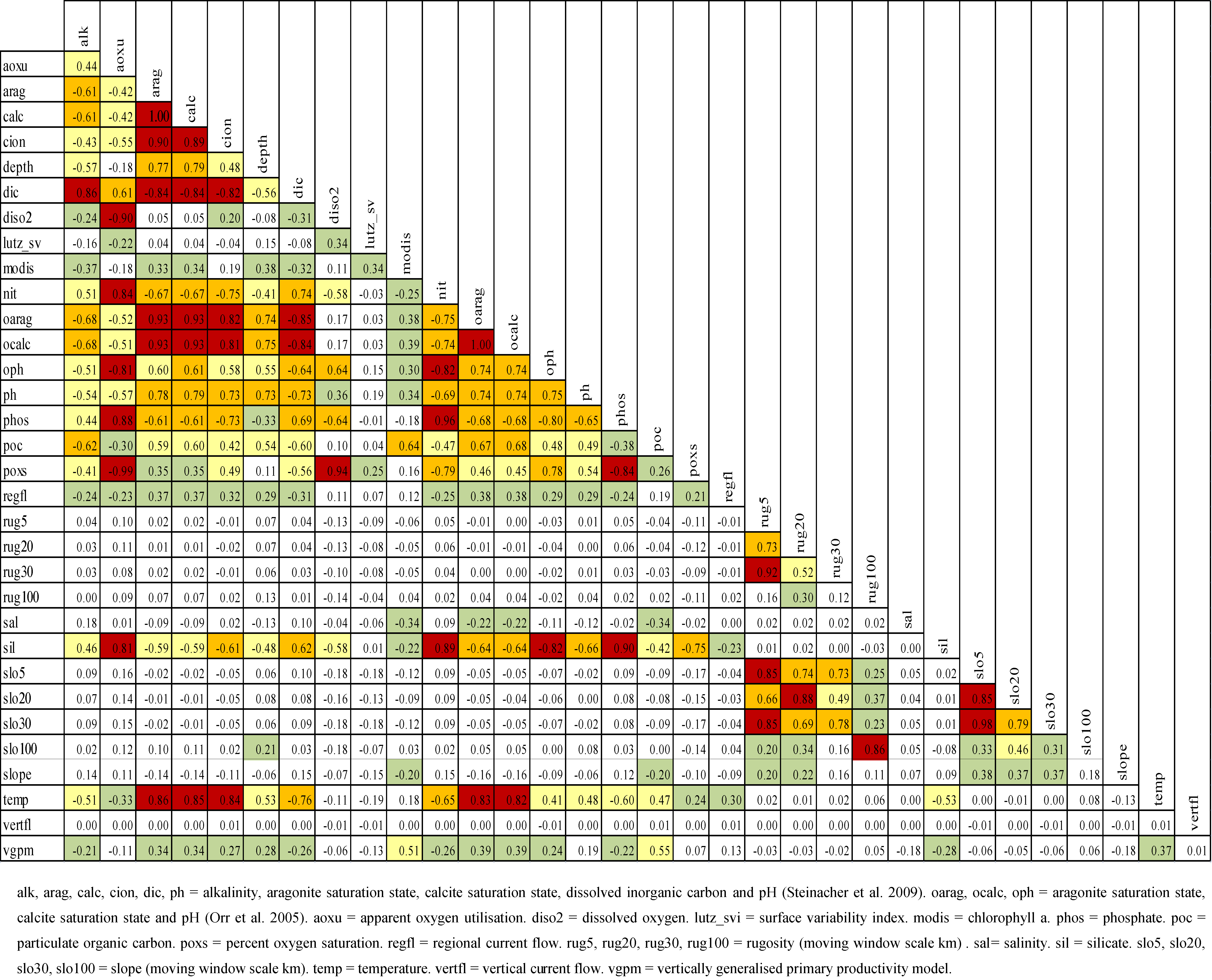

Supplement: Figure S1 — Correlation matrix of the environmental layers developed for this study based on 10,000 randomly distributed points throughout maximum extents (all values significant at p<0.05, Pearson's correlation coefficient). Colour represents correlation strength; no colour = 0–0.2, green = 0.2–0.4, yellow = 0.4–0.6, orange = 0.6–0.8 and red = 0.8–1. The negative sign in a cell represents a negative correlation between the variables, no sign denotes positive. (TIF) [file pone.0018483.s001.tif]

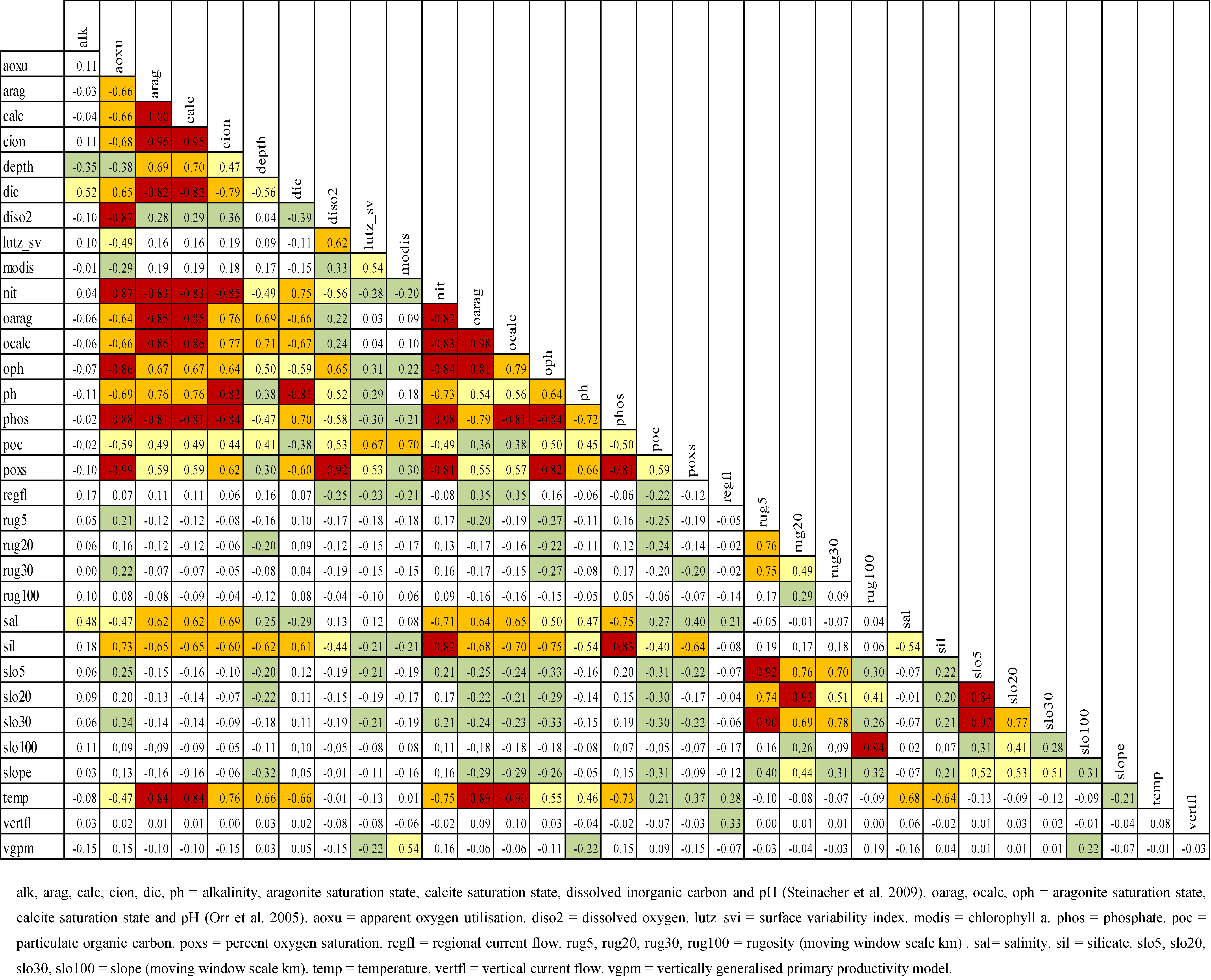

Supplement: Figure S2 — Correlation matrix of the environmental layers developed for this study based upon the species location data (all values significant at p<0.05, Pearson's correlation coefficient). Colour represents correlation strength; no colour = 0–0.2, green = 0.2–0.4, yellow = 0.4–0.6, orange = 0.6–0.8 and red = 0.8–1. The negative sign in a cell represents a negative correlation between the variables, no sign denotes positive. (TIF) [file pone.0018483.s002.tif]

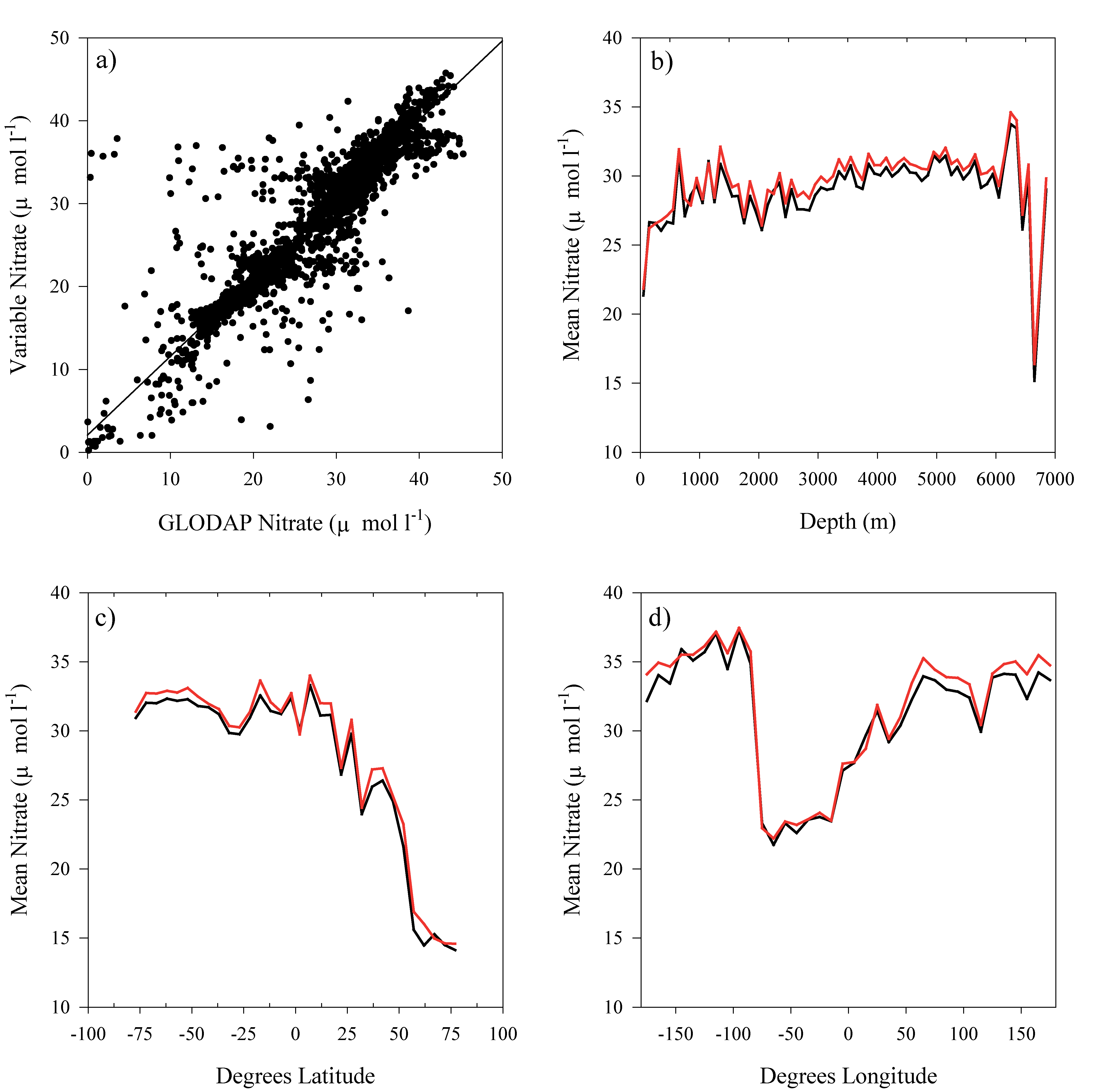

Supplement: Figure S3 — Validation of the environmental layer creation process for nitrate. a) Correlation (0.913) of intersected GLODAP stations with the layer, b) mean nitrate relationships at depth in 50 m bins, c) mean nitrate at latitude in 5° bins and d) mean nitrate at longitude in 10° bins. The black lines are nitrate at each GLODAP bottle station; the red lines are the value of the environmental layer at the position of each GLODAP station. (TIF) [file pone.0018483.s003.tif]

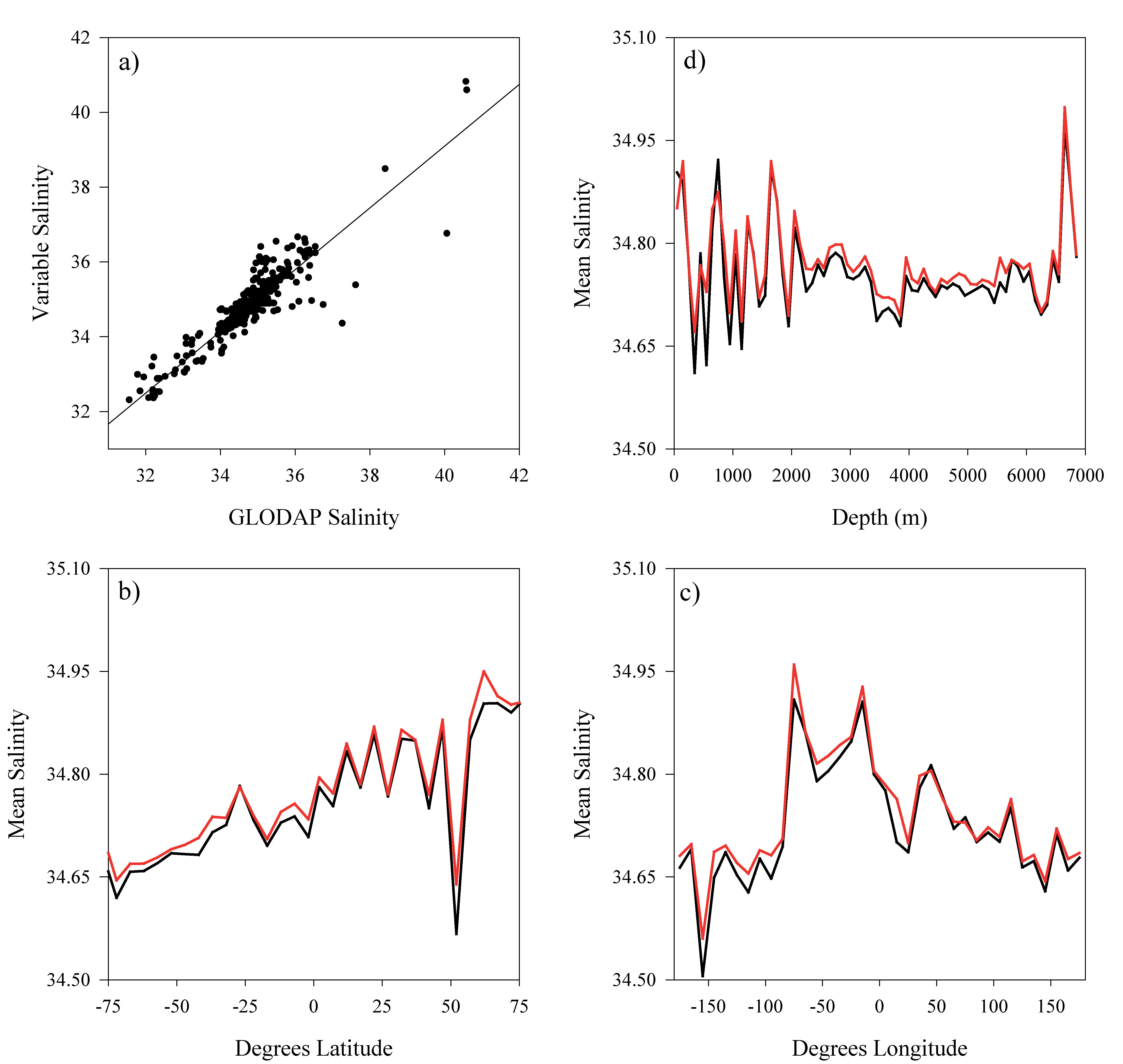

Supplement: Figure S4 — Validation of the environmental layer creation process for phosphate. a) Correlation (0.923) of intersected GLODAP stations with the layer, b) mean phosphate relationships at depth in 50 m bins, c) mean phosphate at latitude in 5° bins and d) mean phosphate at longitude in 10° bins. The black lines are phosphate at each GLODAP bottle station; the red lines are the value of the environmental layer at the position of each GLODAP station. (TIF) [file pone.0018483.s004.tif]

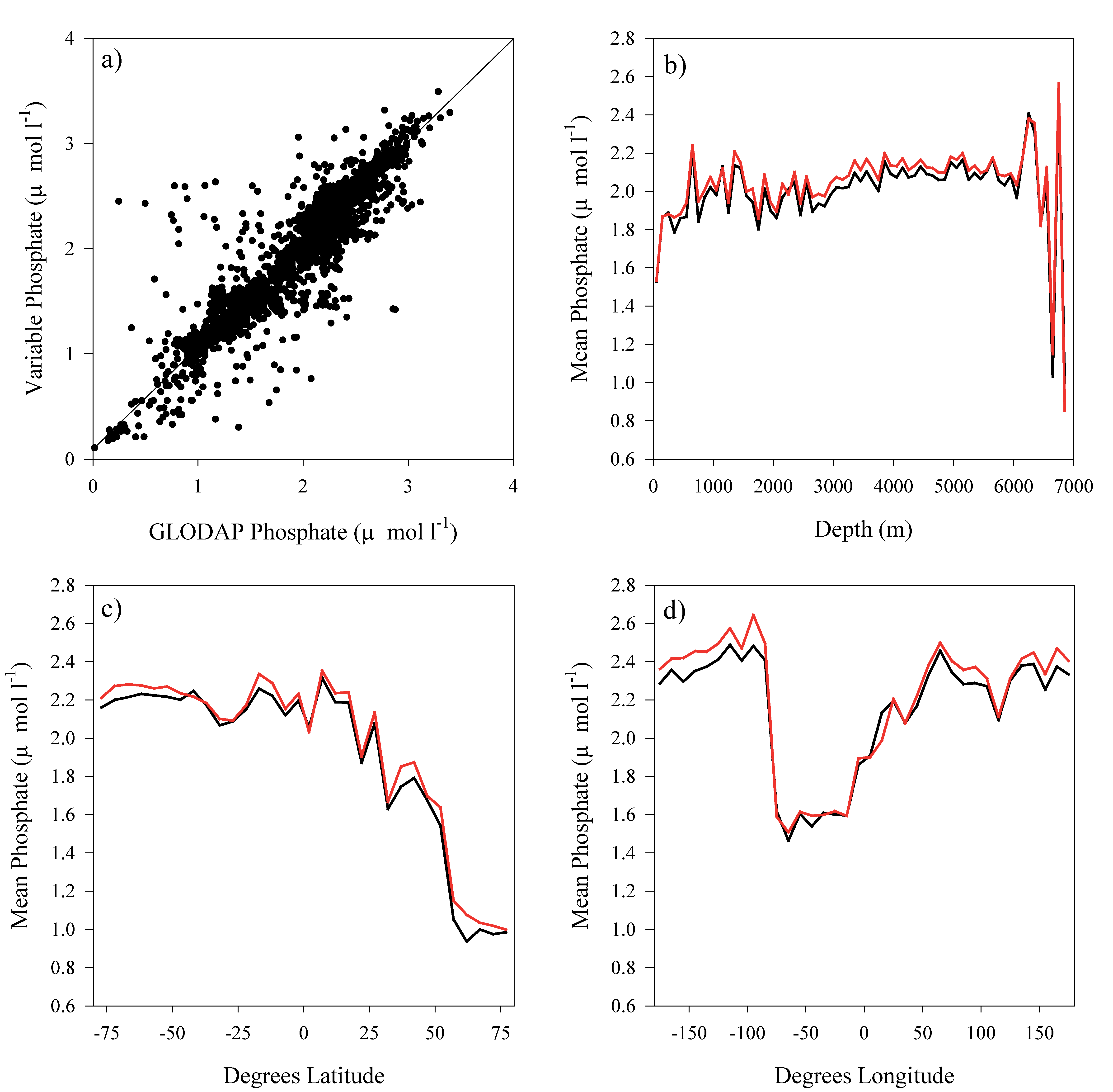

Supplement: Figure S5 — Validation of the environmental layer creation process for salinity. a) Correlation (0.914) of intersected GLODAP stations with the layer, b) mean salinity relationships at depth in 50 m bins, c) mean salinity at latitude in 5° bins and d) mean salinity at longitude in 10° bins. The black lines are salinity at each GLODAP bottle station; the red lines are the value of the environmental layer at the position of each GLODAP station. (TIF) [file pone.0018483.s005.tif]

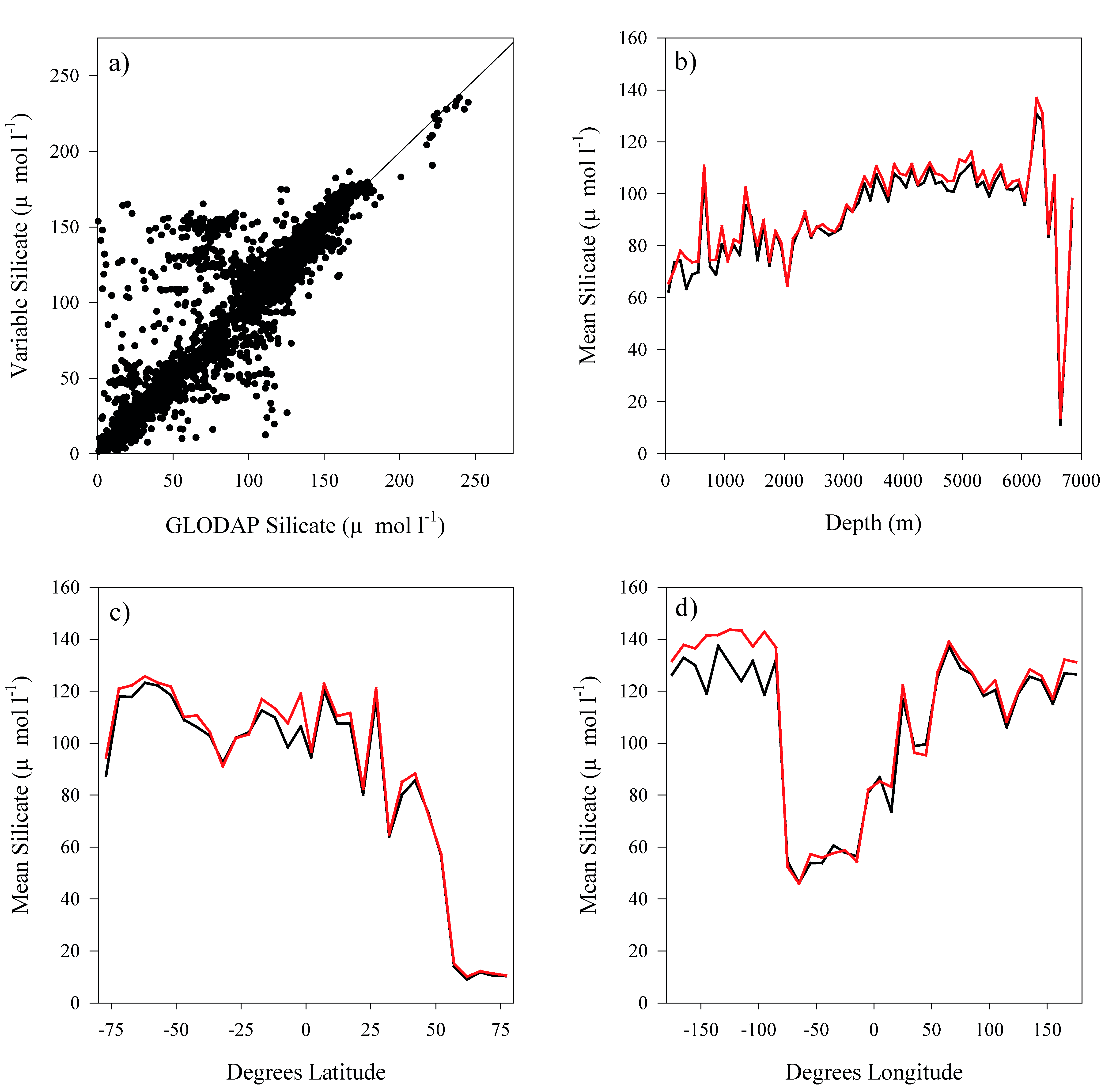

Supplement: Figure S6 — Validation of the environmental layer creation process for silicate. a) Correlation (0.823) of intersected GLODAP stations with the layer, b) mean silicate relationships at depth in 50 m bins, c) mean silicate at latitude in 5° bins and d) mean silicate at longitude in 10° bins. The black lines are silicate at each GLODAP bottle station; the red lines are the value of the environmental layer at the position of each GLODAP station. (TIF) [file pone.0018483.s006.tif]
