# Supplementary material for: Global Habitat Suitability for Framework-Forming Cold-Water Corals
Source: PLoS One. 2011 Apr 15;6(4):e18483. doi: 10.1371/journal.pone.0018483 (PMC3078123; doi:10.1371/journal.pone.0018483)
Supplement: Table S1 — Sources of species locality records that were utilised to develop the presence only dataset for this study. Several datasets contained identical records, which were removed, significantly lowering the number of presences available. Further records removed were, those that fell outside the analysis extent and duplicate records that fell inside a single grid cell. (DOCX) [file pone.0018483.s013.docx]

**Table S1**

|  | *Species* | | | | |  |
| --- | --- | --- | --- | --- | --- | --- |
| Source | *E. rostrata* | *G. dumosa* | *L. pertusa* | *M. oculata* | *S. variabilis* | **Total** |
| HERMES / HERMIONE Programme^1^ | 20 | 0 | 1,859 | 789 | 0 | **2,668** |
| Ocean Biogeographic Information System^2^ | 309 | 363 | 3,094 | 1,330 | 447 | **5,543** |
| Tracey et al (submitted)^3^ | 97 | 204 | 0 | 116 | 191 | **608** |
| United Nations Environment Programme^4^ | 54 | 131 | 2,299 | 580 | 194 | **3,258** |
| United States Geological Survey^5^ | 2 | 0 | 125 | 47 | 6 | **180** |
| Total per species | **482** | **698** | **7,377** | **2,862** | **838** | **12,257** |
| Total after duplicate removal | **215** | **230** | **863** | **591** | **380** | **2,279** |

^1^De Mol, Ben (2009): The HERMES cold-water coral database. Univ Barcelona, Unpublished dataset #728313.

^2^Downloaded from iOBIS.org [01/02/2011] and include the following databases: Azooxanthellate Scleractinia Brazil 01, Benthic biodiversity along the central coast in the Brazilian EEZ (OBIS South America, BRAZIL), Benthic species from the tropical Pacific surrounding New Caledonia, Biodiversity of the Gulf of Mexico Database (BioGoMx), Bishop Museum Data (OBIS distribution), ChEssBase, Cold Water Corals, Hexacorallians of the World, Ifremer BIOCEAN database (Deep Sea Benthic Fauna), IndOBIS, Indian Ocean Node of OBIS, MAR-ECO 2004, Marine and Coastal Research Institute – INVEMAR Colombia IABIN, MV Marine Invertebrates, SeamountsOnline (Seamount Biota), SeSaM, South Western Pacific Regional OBIS Data All Sea Bio Subset.

^3^Tracey DM, Rowden AA, Mackay KA, Compton T. (submitted) Habitat-forming cold-water corals show affinity for seamounts in the New Zealand region.

^4^Cold-water coral records were extracted from version 2.0 of the global point dataset compiled by the UNEP World Conservation Monitoring Centre (UNEP-WCMC), 2005. Sourced from A. Freiwald, Alex Rogers and Jason Hall-Spencer, and other contributors.

^5^Scanlon KM, Waller RG, Sirotek A, Knisel JM, Alesandrini SM. (2010) USGS Cold-water Coral Geographic Database – Gulf of Mexico and Western North Atlantic, Version 1.0. USGS Open File Report.
