# Supplementary material for: Global Habitat Suitability for Framework-Forming Cold-Water Corals
Source: PLoS One. 2011 Apr 15;6(4):e18483. doi: 10.1371/journal.pone.0018483 (PMC3078123; doi:10.1371/journal.pone.0018483)
Supplement: Table S2 — Mean values of the cells of each environmental variable used in the models where species presences were found (standard deviation in parentheses). (DOCX) [file pone.0018483.s014.docx]

**Table S2**

| Species | Depth  (m) | Dissolved oxygen  (ml l^-1^) | Aragonite saturation  state (Ω_ARAG_) | Phosphate  (µmol l^-1^) | Particulate organic  carbon (g C_org_ m^-2^ yr^-1^) | Salinity  (pss) | Slope  (° over 100km) | Temperature  (°C) |
| --- | --- | --- | --- | --- | --- | --- | --- | --- |
| *E. rostrata* | -1021.78  (786.41) | 4.24  (1.00) | 1.42  (0.65) | 1.71  (0.67) | 6.97  (5.94) | 34.72  (0.47) | 0.85  (0.61) | 7.54  (4.97) |
| *G. dumosa* | -543.38  (365.39) | 5.22  (0.61) | 1.73  (0.35) | 1.34  (0.42) | 21.83  (9.99) | 34.60  (0.19) | 0.43  (0.40) | 8.32  (2.24) |
| *L. pertusa* | -726.53  (647.33) | 4.90  (1.03) | 1.84  (0.45) | 1.06  (0.39) | 26.25  (20.92) | 35.36  (0.34) | 0.66  (0.58) | 9.39  (2.95) |
| *M. oculata* | -857.73  (698.98) | 4.35  (1.02) | 1.64  (0.56) | 1.37  (0.60) | 15.03  (13.63) | 35.15  (0.50) | 0.78  (0.61) | 8.90  (3.93) |
| *S. variabilis* | -1305.73  (746.09) | 4.61  (0.82) | 1.22  (0.44) | 1.81  (0.56) | 9.10  (6.88) | 34.75  (0.47) | 0.81  (0.61) | 5.37  (3.59) |
